# Water Productivity Mapping (WPM) Using Landsat ETM+ Data for the Irrigated Croplands of the Syrdarya River Basin in Central Asia

**DOI:** 10.3390/s8128156

**Published:** 2008-12-10

**Authors:** Alexander Platonov, Prasad S. Thenkabail, Chandrashekhar M. Biradar, Xueliang Cai, Muralikrishna Gumma, Venkateswarlu Dheeravath, Yafit Cohen, Victor Alchanatis, Naftali Goldshlager, Eyal Ben-Dor, Jagath Vithanage, Herath Manthrithilake, Shavkat Kendjabaev, Sabirjan Isaev

**Affiliations:** 1 International Water Management Institute (IWMI), 127, Sunil Mawatha, Colombo, Sri Lanka; 2 U.S. Geological Survey, 2255 N. Gemini Drive, Flagstaff, AZ 86001, USA; 3 University of Oklahoma, 101 David L. Boren Blvd, Norman, Oklahoma 73019, USA; 4 United Nations Joint Logistics Center, Juba, Sudan; 5 Institute of Agricultural Engineering, ARO; 6 University of Soil Sciences, ARO, Volcani center, Bet Dagan 50250, Israel; 7 Department of Geography, P.O. B. 39040, Tel-Aviv University 69989, Israel; 8 Central Asian Scientific Research Institute of Irrigation, Block 11, Karasu-4, Tashkent, 700187, Uzbekistan; 9 Scientific Research Institute for Growing Cotton, Uzbekistan, Tashkent; 10 International Water Management Institute (IWMI), Apt. 123, House 6, Murtazaeva Street, Tashkent 700000, Uzbekistan

**Keywords:** Water productivity mapping, remote sensing, water use, crop productivity, crop yield modeling, simplified surface energy balance model, Central Asia, Syrdarya river basin

## Abstract

The overarching goal of this paper was to espouse methods and protocols for water productivity mapping (WPM) using high spatial resolution Landsat remote sensing data. In a world where land and water for agriculture are becoming increasingly scarce, growing “more crop per drop” (increasing water productivity) becomes crucial for food security of future generations. The study used time-series Landsat ETM+ data to produce WPMs of irrigated crops, with emphasis on cotton in the Galaba study area in the Syrdarya river basin of Central Asia. The WPM methods and protocols using remote sensing data consisted of: (1) crop productivity (ton/ha) maps (CPMs) involving crop type classification, crop yield and biophysical modeling, and extrapolating yield models to larger areas using remotely sensed data; (2) crop water use (m^3^/ha) maps (WUMs) (or actual seasonal evapotranspiration or actual ET) developed through Simplified Surface Energy Balance (SSEB) model; and (3) water productivity (kg/m^3^) maps (WPMs) produced by dividing raster layers of CPMs by WUMs. The SSEB model calculated WUMs (actual ET) by multiplying the ET fraction by reference ET. The ET fraction was determined using Landsat thermal imagery by selecting the “hot” pixels (zero ET) and “cold” pixels (maximum ET). The grass reference ET was calculated by FAO Penman-Monteith method using meteorological data. The WPMs for the Galaba study area demonstrated a wide variations (0-0.54 kg/m^3^) in water productivity of cotton fields with overwhelming proportion (87%) of the area having WP less than 0.30 kg/m^3^, 11% of the area having WP in range of 0.30-0.36 kg/m^3^, and only 2% of the area with WP greater than 0.36 kg/m^3^. These results clearly imply that there are opportunities for significant WP increases in overwhelming proportion of the existing croplands. The areas of low WP are spatially pin-pointed and can be used as focus for WP improvements through better land and water management practices.

## 1. Introduction

Increasing water scarcity and competition for the water and land from agricultural and non-agricultural sectors drive the need to improve crop water productivity to guarantee adequate food for future generations with the same or less water and land than that is currently available for agriculture [1-3]. Increasing water productivity can be important pathway for poverty reduction, especially in developing countries, where the variability of water productivity of within and between fields is very high, according to the specific conditions under which the crop are grown [4].

The crop water productivity is a vital parameter to assess the performance of irrigated and rainfed agriculture [5], it can be represented in physical or economic units [6]. The physical crop water productivity (kg/m^3^) is the ratio of crop yield (ton/ha) to the amount of water used (m^3^/ha). The economic water productivity ($/m^3^) relates the economic benefits per unit of water used. Water productivity studies at different scales are the direction of investigation of many researchers in the world, but existing studies [7-10] are predominantly dependent on non-remote sensing approaches, based on capturing point data and/or official statistics.

In recent years several remote sensing methods have been developed (SEBAL, METRIC), which contributed valuable data for irrigation management. Time-series remote sensing data in optical and thermal bands provides an excellent opportunity to understand and map water productivity (kg/m^3^) over large areas [11]. Usually the low spatial resolution satellite images (NOAA AVHRR, MODIS) were the source for water use and water productivity analysis in the world [12-16]. A limited number of such studies used the high spatial resolution remote sensing data [17-22], but not in Central Asia region.

Given the above background, the overwhelming emphasis of this study was to develop comprehensive sets of simplified methods and protocols for water productivity mapping (WPM) using high spatial resolution satellite images (Landsat ETM+), based on combination of crop classification, modeling of crop yield, and water use (actual evapotranspiration) computation for crops using thermal band data and surface energy balance models.

The study used satellite images for the year 2006 and focused on WPMs of irrigated cotton fields inside the Galaba study area of the Syrdarya river basin, in Uzbekistan, Central Asia.

## 2. Study area

**Figure 1.**
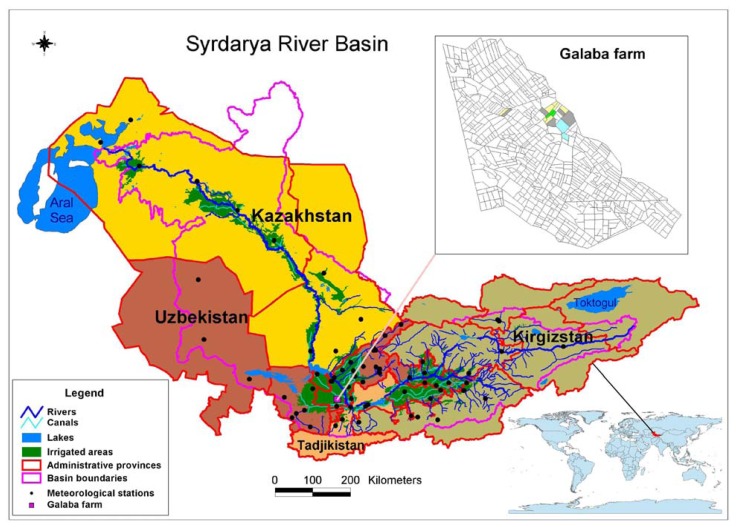
Location of the study area.

The study area (Galaba farm) is located in the middle part of the Syrdarya river basin (Figure 1). The climate at the study area is sharply arid, the low amount of precipitation (260-320 mm/year) occurs in autumn-winter-spring time, with common maximum in March. The coldest month is January, when monthly average air temperature varies from 0-13 °C. The absolute minimum temperature is not lower than -18 °C. The highest duration of solar hours is in summer months, reaching up to 295-390 hours per month.

The soil of Galaba farm is representative for the Syrdarya province: heavy loamy soil, and variable soil salinity. A numerous sample site locations were selected to capture within and between fields spatial variability of vegetation condition for different crops (winter wheat, cotton, rice and maize) inside the Galaba study area. The regular phenological observations and crop yield measurements at harvest time were made during the 2006 crop growing season.

## 3. Processing of satellite images

A series of Landsat-7 ETM+ satellite images (Table 1) were acquired and used in this study. The SLC-off gaps were filled by image provider. The images cover the cropping season and have a frequency of about one image per month. Each image was georeferenced to WGS-84 coordinate system by using, as a reference, the GeoCover products [23].

The use of gap filled images is justified due to several reasons. First, the study area (Galaba farm) is located in the center of images, so the gap filling pixels are an insignificant proportion of the total image area. Second, the Landsat-7 ETM+ images (after May, 2003) comes with the gap masks for each band and only non-gap filled part of images were used in this research to develop yield models. Third, once the yield model were developed using only non gap filled data, the spatial extrapolation of the model were performed using complete image data (gap filled portion included) for completeness. Fourth, one of the best method for SLC-off gap filling was used and this involved using the nearest possible dates to maintain spectral integrity of multi-temporal data. Fifth, the Landsat is the only satellite providing the high spatial resolution thermal imagery (except ASTER which is highly infrequent). These factors highlight the use of gap-filled images and on how best to use them to avoid the effects of gap-filling.

### 3.1 Digital number to radiance

The Landsat ETM+ 8 bit digital numbers were converted to radiances using the equation:

(1) $$L_{\lambda }=\text{gain}*DN+\text{offset}$$

This can also be expressed as:

(2) $$L_{\lambda }=\frac{\text{LMAX}-\text{LMIN}}{DN\_\text{MAX}-DN\_\text{MIN}}*(DN-DN\_\text{MIN})+\text{LMIN}$$

where: L_λ_ is the radiance (W m^-2^ sr^-1^ μm^-1^), *DN_MIN* = 1, *DN_MAX* = 255, and *DN* are the digital number of pixels, *LMIN* and *LMAX* are the spectral radiances (W m^-2^ sr^-1^ μm^-1^) for each band at *DN_MIN* and *DN_MAX,* respectively are presented in Table 2.

### 3.2 Radiance to reflectance

A reduction in between-scene variability can be achieved through a normalization for solar irradiance by converting spectral radiance, as calculated above, to planetary reflectance or albedo [24, 25]. This combined surface and atmospheric reflectance of the Earth is computed with the following formula:

(3) $$\rho_{\text{p}}=\frac{\pi *L_{\lambda }*d^{2}}{{\text{ESUN}}_{\lambda }*\cos\theta_{s}}$$

where: ρ_p_ is the at-satellite exo-atmospheric reflectance (percentage), L_λ_ is the radiance (W m^-2^ sr^-1^ μm^-1^), d is the earth to sun distance in astronomic units at the acquisition date [25], ESUN_λ_ is the mean solar exo-atmospheric irradiance or solar flux (W m^-2^ sr^-1^ μm^-1^) [26], and *θ_S_* is solar zenith angle in degrees (*i.e.;* 90 degrees minus the sun elevation or sun angle when the scene was recorded as given in the image header file).

### 3.3 At-sensor temperature from thermal bands

Landsat produces two thermal images, one using a low gain setting (Band 6L), saturated at 347.5 K and other using a high gain setting (Band 6H), saturated at 322 K. Usually Band 6H is used for analysis of vegetated surfaces. The thermal band converted to at-sensor (satellite) radiance using equation (2) and to at-satellite temperature using the formula:

(4) $${\text{T}=\text{K2}/\text{Ln}\;(\text{K1}/\text{L}}_{\lambda }+\text{1})$$

where: T - at-sensor (satellite) temperature in degree Kelvin

K2 – calibration constant, (for Landsat-7: K2 = 1282.71)
K1 – calibration constant, (for Landsat-7: K1 = 666.09)
L_λ_ – spectral radiance in W m^-2^ sr^-^^1^ μm^-1^

### 3.4 Normalized difference vegetation index (NDVI)

A widely used vegetation index is Normalized Difference Vegetation Index (NDVI), which defined as the ratio:

(5) NDVI=(NIR−Red)/(NIR+Red)

where: *NIR* and *Red* are the spectral reflectance of the vegetated land surface in the near infrared (Band 4) and red (Band 3) Landsat bands, respectively [27].

## 4. Field-plot data characteristics

Field-plot data were acquired to correspond with the satellite sensor overpass dates (Table 1). Data from 273 field-plot points were gathered during the 2006 year. The data consisted of biophysical parameters such as wet and dry biomass (kg/m^2^), crop yield (ton/ha) and leaf area index (LAI) (m^2^/m^2^) measurements by AccuPAR LP-80 ceptometer [28]. Meteorological data (air temperature, relative humidity, solar radiation, wind speed and rainfall) were measured by a WatchDog weather station [29], installed inside Galaba farm. Enough care has been taken while selecting the location of the trial field-plots (replications) to represent the between-fields variability of crops condition across the farm. Usually the plant samples, together with other measurements, were taken every 15 days inside each test field of Galaba farm for the four main crops: cotton (5 fields), wheat (4 fields), rice (2 fields), and maize (2 fields). The average values of various variables collected from the field-plots are shown in Table 3.

The local variety of cotton (E4727) was sown in all cotton test fields, the sowing date varied from 6 of April, to 16 of May. The date of first harvesting was in range (9-16) of September and second (final) pick-up was made at 30 of September. The cotton yield from each test field at 3-5 locations was measured by lint harvesting from the area of 10 m^2^ and the samples weighting.

## 5. Methods

The applied methodology of crop water productivity mapping (WPM) consists of the following steps:

1. Crop productivity mapping (CPM);
2. Water use (actual evapotranspiration) mapping (WUM); and
3. Water productivity mapping (WPM).

### 5.1 Crop productivity maps (CPMs)

The CPMs were produced for specific crops. First, this required precise delineation of crop types (section 5.1.1). Second, the field measured yield quantities were related to spectral indices and wavebands leading to crop yield models (section 5.1.2). Third, the best yield models were extrapolated to larger area using remotely sensed data to obtain CPMs (section 5.1.3).

#### 5.1.1 Crop type mapping using remote sensing

In this study we use the strength of the temporal data in separating crop types. The normalized difference vegetation index (NDVI) raster layers, created from six Landsat-7 ETM+ images give possibility to analyze temporal NDVI changes for pixels inside the farm fields of Galaba study areas with sown crops. By overlaying of polygon vector GIS layer of fields with NDVI raster layers the average NDVI values for each field were calculated, which were saved in the attribute table of geographic information systems (GIS) layer using ArcView software [30].

#### 5.1.2 Crop yield modeling by relating remote sensing indices with field-measured **yield**

Remote sensing has proved very useful in estimating crop yields [18, 31-33] and the relationships have improved with the use of modern high spectral and spatial resolution sensors [34-39]. For example, the Normalized Difference Vegetation Index (NDVI) was found to correlate with net primary production, biomass, vegetation fraction, and yield [40-43].

The crop biophysical and yield variables were often related to spectral measurements from space. The most commonly used crop variables establishing such relations were the leaf area index (LAI), wet biomass (WBM), dry biomass (DBM), and yield (YLD). When crop yields were not measured, it can be derived using knowledge of the biomass and developing harvest index (yield/biomass) relationships [44, 45]. In this research the main focus was in relating the measured cotton yield at test fields with NDVI derived from Landsat-7 ETM+ images.

#### 5.1.3 Crop productivity maps (CPMs) by applying best yield models to specific crops

One of the biggest strengths of remote sensing lies in the observation of the entire landscape rather than just few points. With good understanding of the relationships between crop yield and vegetation index (section 5.1.2) it is possible to extrapolate the understanding gained through models to larger areas using remotely sensed data of specific crops (section 5.1.1). The approach we employed in this study is listed in the following steps:

(a) measuring crop variables through field campaign (Table 3);
(b) acquiring the images that correspond to field campaign dates (Table 1);
(c) delineating the crop types (section 5.1.1);
(d) developing modes that relate vegetation index with actual crop yield (section 5.1.2); and
(e) extrapolating the best models to larger areas using remotely sensed data (section 5.1.3).

### 5.2 Water use (actual evapotranspiration) map

Water used by crops (in m^3^/ha or mm/m^2^) was determined from remote sensing by calculating the actual ET based on the following steps:

- determining the ET fraction from Landsat ETM+ thermal data;
- calculating the reference ET by applying Penman-Monteith equations; and
- computing the actual ET by multiplying ET fraction with reference ET.

#### 5.2.1 Modeling of ET fraction (crop coefficients) by SSEB model

The ET fraction [46] or evaporative fraction [47, 48] is the ratio of actual ET over reference ET. The ET fraction was calculated by the Simplified Surface Energy Balance (SSEB) model, described in Senay *et al.* [46], based on assumption, that the latent heat flux (actual ET) varies linearly between the land surface temperature (LST) of “hot” and “cold” pixels:

(6) $${\text{ET}}_{\text{fraction}}=({\text{T}}_{\text{hot}}-\text{T})/({\text{T}}_{\text{hot}}{-\text{T}}_{\text{cold}})$$

where: ET_fraction_ is the fraction of ET (dimensionless); T is the land surface temperature (LST) of any pixel; T_hot_ and T_cold_ are the LST of “hot” and “cold” pixels respectively, the LST expressed in degree Kelvin or Celsius. The “hot” and the “cold” pixels are selected inside the irrigated fields of the investigated area for each image.

#### 5.2.2 Calculation of the reference ET

The reference ET (or “potential ET: term used before creating of terminology standard) can be calculated from meteorological data using a number of (semi-) empirical equations by different methods (Priestley-Taylor, Blaney-Criddle, Hargreaves, Penman, Penman-Monteith). The comparison of methods is briefly provided by Kassam *et al.* [49] and Wright *et al* [50, 51]. Some methods are applicable for only specific climatic conditions, humid or arid [52].

To separate the influence of the weather conditions on the evapotranspiration, the concept of reference ET, as the evapotranspiration from reference crop, grown in ideal conditions (disease-free, well-fertilized, under optimum soil water content), having the fixed parameters. There are two well known methods for reference ET calculations:

- **FAO method**: Allen *et al* [52] recommended to use the clipped grass (hypothetical crop) having the plant height of 0.12 m, a surface resistance of 70 s m^-1^ and an albedo of 0.23 for reference ET (ETo) calculation.
- **ASCE Method**: The ASCE-PM method uses as a reference the alfalfa crop of 0.5 m plant height, albedo of 0.23, but different surface resistance of 50 s m^-1^ at daytime and 200 s m^-1^ at nighttime, for reference ET (ET_r_) calculation. The method compares well against lysimeter measurements of alfalfa ET at Kimberly, Idaho [50] and at Bushland, Texas [53].

The ASCE-EWRI [54] standardized the Penman-Monteith method for reference ET calculation from meteorological data for either alfalfa or grass reference by formula (7).

(7) $$ET\_\text{ref}=\frac{0.408*\Delta *(R_{n}-G)+\gamma *\frac{Cn}{T+273}*u_{2}*(e_{s}-e_{a})}{\Delta +\gamma *(1+Cd*u_{2})}$$

where: ET_ref - the reference evapotranspiration [mm day^-1^],

- R_n_ - the net radiation at the crop surface [MJ m-2 day^-1^],
- G - the soil heat flux density [MJ m^-2^ day^-1^],
- T - the mean daily air temperature at 2 m height [°C],
- u_2_ - the wind speed at 2 m height [m s-1],
- e_s_ - the saturation vapour pressure [kPa],
- ea - the actual vapour pressure [kPa],
- (e_s_ - e_a_) - the saturation vapour pressure deficit [kPa],
- Δ_ _ - the slope vapour pressure curve [kPa °C^-1^],
- γ_ - the psychrometric constant [kPa C^-1^].

Cn (K mm s^3^ Mg^-1^ d^-1^ or K mm s^3^ Mg^-1^ h^-1^) and Cd (s m^-1^) are the constant, that changes with reference type and calculation time step (Table 4), adapted from Allen *et al.* [17].

The crop evapotranspiration (ET_c_) is determined by multiplying the grass reference evapotranspiration (ETo) by the crop coefficient (Kc). This will be the evapotranspiration from disease-free, well-fertilized crops, grown in large fields, under optimum soil water content [52]:

(8) $${\text{ET}}_{\text{c}}={\text{Kc}*\text{ET}}_{\text{o}}$$

The crop coefficient (Kc) integrated the differences of field crops at different stages of growth from the reference surface having fixed crop height, surface resistance, and albedo. The Kc can be separated into two coefficients: a basal crop (Kcb) and soil evaporation coefficient (Ke), so called “dual” crop coefficient approach [52, 55]:

(9) Kc=Kcb+Ke

The basal crop coefficient (Kcb) represents the transpiration part of ETc - the ratio of the crop evapotranspiration (ETc) over the reference evapotranspiration (ETo), when the soil surface is dry but transpiration occurred at potential rate, without water limitation. The soil evaporation coefficient (Ke) represents the evaporation part of ETc, after soil wetting by precipitation or irrigation.

#### 5.2.3 Calculation of actual seasonal ET (water use) for selected crops

The amount of water used by crops is equal to actual seasonal evapotranspiration (ET_actual_). The evapotranspiration combines two separate processes: the evaporation from the soil surface (or water) and transpiration from the vegetation. The driving force of evaporation is the difference between the water vapour pressure at the evaporating surface and the air. The transpiration consists of liquid water vaporization from vegetation leaves through stomata. This water is taken by the roots from soil and transported through the plant.

Both processes depend on the solar radiation supply, the vapour pressure gradient and wind speed, but transpiration is also influenced by crop condition (type, variety and development stage, plant density), environmental conditions (soil salinity, fertility and texture, amount of fertilizers and pests) and crop cultivation practices (soil water content).

During the vegetation growth the fraction of evaporation/transpiration in evapotranspiration keeps changing. At the early stage of growth, the soil evaporation is dominated, but when the vegetation covers the soil, the transpiration becomes the main process.

The ET_actual_ based on remote sensing data is calculated by multiplying the ET fraction (ET_fraction_) with grass reference ET (ET_o_):

(10) $${\text{ET}}_{\text{actual}}={\text{ET}}_{\text{fraction}}*{\text{ET}}_{\text{o}}$$

In recent years several methods have been developed for modeling actual ET from satellite images, which contributed valuable data for irrigation management.

First, is the Surface Energy Balance Algorithm for Land (SEBAL) method, described in Bastiaanssen *et al.* [47]. It uses the images that record a visible, infrared, and thermal infrared radiation data from satellites such as Landsat, ASTER, MODIS, NOAA AVHRR). Actual ET is computed on a pixel-by-pixel basis for the instantaneous time of the satellite image, as the residual amount of energy remaining from the classical energy balance:

(11) λET=Rn−G−H

where: λET is latent heat flux (the energy used for evapotranspiration), Rn is net radiation at the surface, G is soil heat flux, and H is sensible heat flux to the air. All fluxes in W m^-2^ day^-1^ units.

ET (mm day^-1^) is calculated from latent heat flux by dividing it by the latent heat of water vaporization (λ). SEBAL is used in different parts of the world. Validation of SEBAL has been reported by Bastiaanssen [56, 57] and Tasumi [58].

Second, is the Mapping EvapoTranspiration with high Resolution and Internalized Calibration (METRIC) method described in Allen *et al.* [48]. METRIC uses the SEBAL approach for estimating the near surface temperature gradient, as a function of radiometric surface temperature and internal calibration at the “hot” and “cold” pixels of the sensible heat computation. METRIC calibrated to each satellite image by using of alfalfa, as the reference crop, because this crop more common for USA condition. According to Allen *et al.* [59], the actual ET, calculated by METRIC, has very high correlation with ET, measured by lysimeters. The SEBAL and METRIC methods based on linear relationship between the near-surface temperature difference and the land surface temperature for sensible heat flux estimation, by assuming that the “hot” pixels have no latent heat (ET = 0) and the “cold” pixels have maximum ET.

Using these methods require a solid knowledge of energy balance, radiation physics, vegetation parameters, and weather data. The methods based on theoretical and physical relationships, but include the empirical coefficients, which must be calibrated for local conditions [60].

The SSEB method adopted in this paper and described in sections 5.2.1 through 5.2.3 significantly simple, when compared with above mentioned models to determine ET_actual_ based on remote sensing. According to Senay *et al.* [46], the correlation coefficient between actual ET from SSEB with METRIC varied from 0.94 to 0.99 and with SEBAL from 0.55 to 0.79, depending on the crop type.

### 5.3 Water productivity mapping (WPM)

The water productivity map (WPM) was created by dividing the crop productivity map (CPM; section 1.1) with water use map (WUM; section 2.2):

(12) WP=(Crop productivity)/(Water use)

where: WP is water productivity (kg/m^3^ or $/m^3^), Crop productivity is crop yield (kg/m^2^ or ton/ha) or economic value ($/ha), Water use is seasonal actual ET (mm, m^3^/m^2^ or m^3^/ha).

## 6. Results and discussions

### 6.1 Crop productivity calculations

#### 6.1.1 Crop type classification

**Figure 3.**
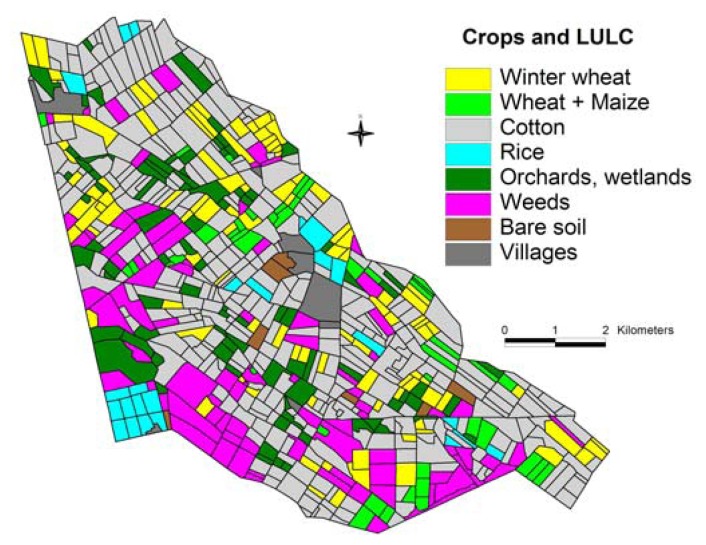
Crop types inside the Galaba farm, derived using time-series Landsat ETM+ images for year 2006.

**Figure 4.**
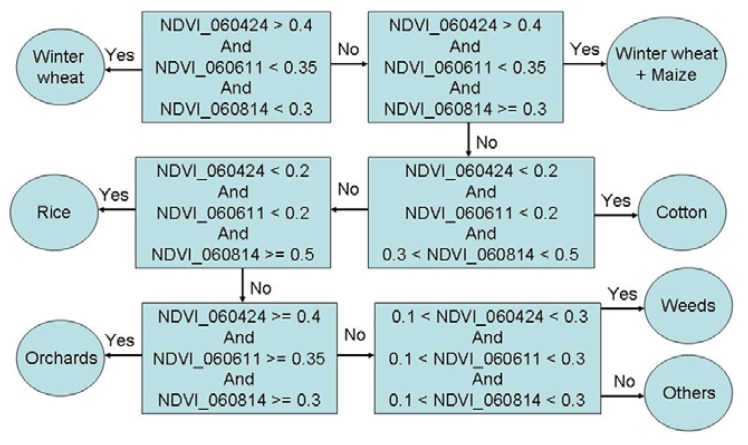
Decision rules for main crops classification.

The crop classification (Figure 3) was made by using NDVI threshold values and applying the decision rules (Figure 4) by “if-else” criteria in ERDAS Imagine [61] modeler.

**Figure 5.**
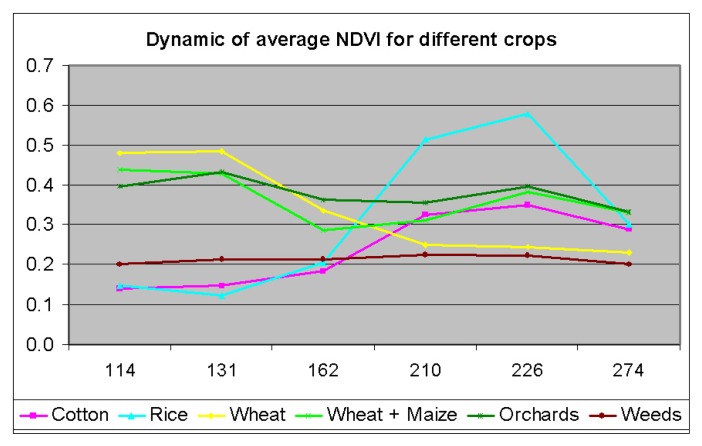
Dynamic of average NDVI for different crops in Galaba study area, derived from Landsat ETM+ images for 2006 year.

The pixels having regularly low NDVI (< 0.2) were assigned the class of bare soil. Rice crop was distinctly different from other crops during day of year (DOY) 210 through 226 (Figure 3). Cotton and rice fields have near similar signatures during DOY 114, 131, 162, and 274, but were distinctly different during DOY 162 and 210 (Figure 5). Cotton was similar to wheat during DOY 210, 226, and 274 but was distinctly different during DOY 114, 131, and 162. Overall, time-series data facilitated differentiating crop types through significant NDVI variations between crop types during at least 3 of the 6 dates (Figure 5). The vector files of road networks and settlements in the Galaba farmland areas were overlaid to delineate them. Any “pepper and noise” within crop fields were smoothed using post processing techniques involving 3×3 window kernel smoothing by spatial modeling in ERDAS Imagine [61]. Given that we had intimate knowledge of the farm fields studied due to repeated visits throughout the growing season, we were able to check the accuracy of classification of farm fields (each field having one crop). The accuracy of main crops classification was perfect, close to 100%.

#### 6.1.2 Crop yield modeling

**Figure 6.**
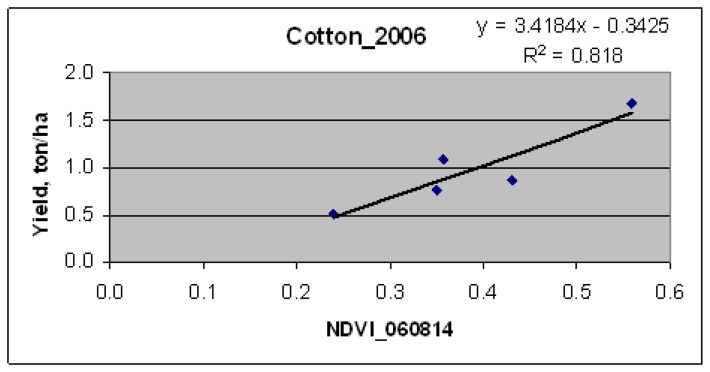
Measured cotton yield versus Landsat ETM+ NDVI relationship from test fields of Galaba study area.

The cotton yield (ton/ha) values, measured at test fields, were correlated with average for these fields NDVI, derived from Landsat ETM+ images [62]. The best relationship (Figure 6a) with an R^2^-value of 0.818 was obtained for image of DOY 226 (14 August, 2006), when cotton was in mid-season growth stage.

#### 6.1.3 Crop productivity calculations by extrapolating to larger areas

**Figure 7.**
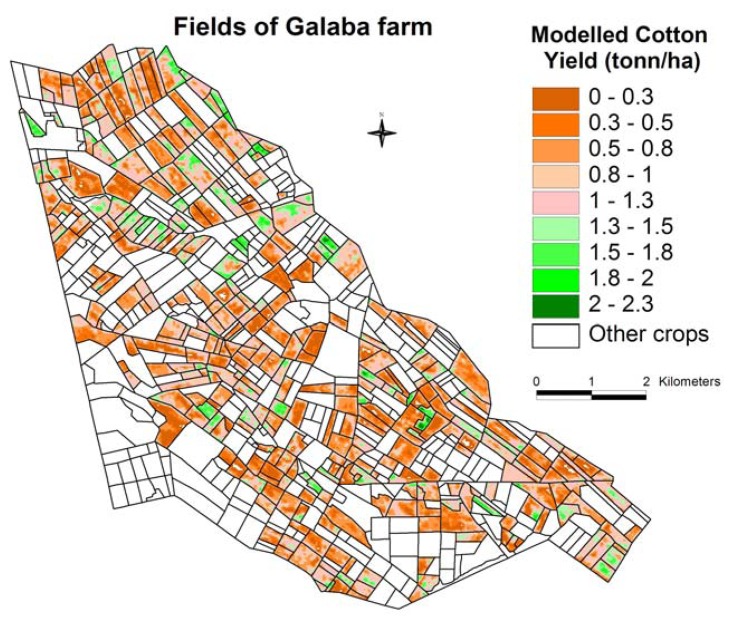
Cotton crop productivity map (CPM) derived from the best NDVI-yield correlation.

The yield model (Figure 6) was applied to all cotton fields (Figure 3) taking the NDVI image on DOY 226. This resulted in an image with cotton yield expressed in ton/ha (Figure 7), The limited number of cotton test fields did not allow us to validate the yield model on other fields, because of budget constraints.

### 6.2 Water use (seasonal ETactual)

#### 6.2.1 Modeling of ET fraction (crop coefficient)

**Figure 8.**
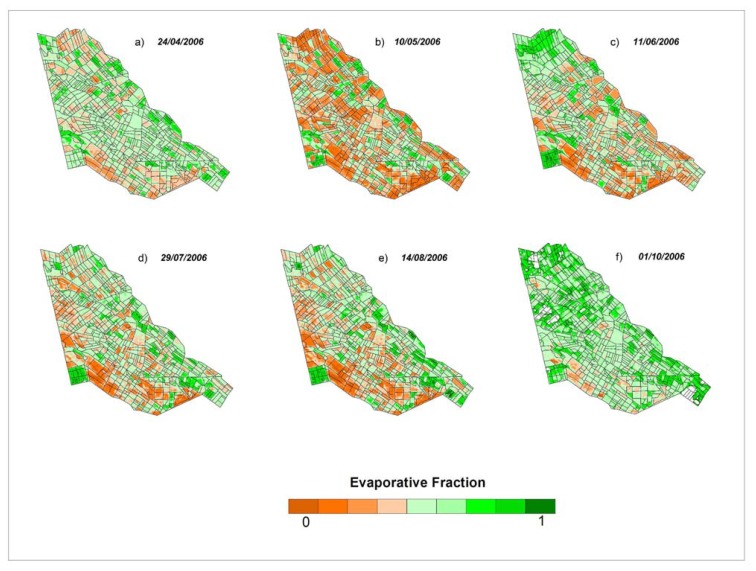
Seasonal changes of ET fraction, derived using thermal band of Landsat ETM+ for the Galaba study area during 2006.

The ET_fraction_ (dimensionless) raster layers (Figure 8) were derived using simplified surface energy balance (SSEB) model (Equation 6).

In April (Figure 8a) winter wheat is in mid-season growth stage and hence we see significant values of ET_fraction_ for this crop. The ET_fraction_ is mostly low in May (Figure 8b) and early June (Figure 8c) because cotton is either just planted or in early growth phases and winter wheat is harvested. The ET_fraction_ increases significantly (green color spreads spatially and increases in intensity) in July (Figure 8d) and reaches almost maximum in August (Figure 8e), when main crops (cotton, rice and maize) are in mid-season growth stages. In the beginning of October (Figure 8f), the main crops were harvested and ET_fraction_ further increased and this can be explained by weeds germination, especially inside the abandoned lands, after rainfall at the end of September. Besides, the October image is contaminated by clouds and its shadow, which reduce the land surface temperature values.

**Figure 9.**
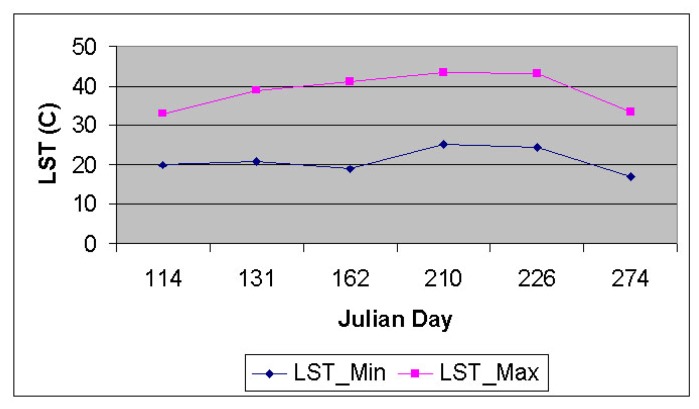
Minimum (cold) and maximum (hot) LST values from six Landsat images.

Enough care is required for selecting the “hot” and the “cold” pixels, which was selected inside the farm fields. The minimum (“cold”) and maximum (“hot”) land surface temperatures from selected pixels inside six Landsat-7 ETM+ images are shown on Figure 9.

**Figure 10.**
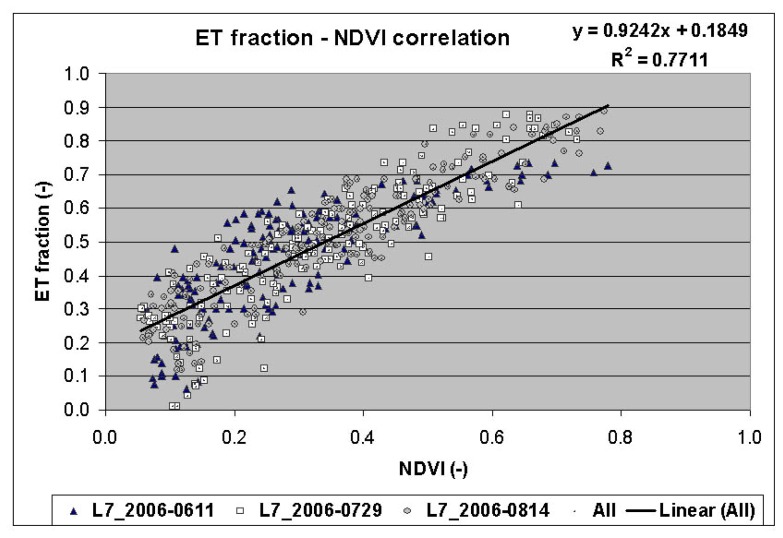
Landsat ETM+ ET fraction versus NDVI relationship.

The ETM+ NDVI values of the cotton crop were related to ET_fraction_ from ETM+ images of the same fields for 3 dates. The results showed a high degree of correlation between NDVI and ET_fraction_ of the Landsat ETM+ with an R^2^ value of 0.6831 for April image, 0.6548 for June image, 0.8472 for August image, and 0.7711 for the pooled data from 3 images (Figure 10). The R^2^ values were lower for images of early date when influence of bare soil on ET was significant and the process of evaporation dominated over transpiration.

#### 6.2.2 Reference ET calculation

**Figure 11.**
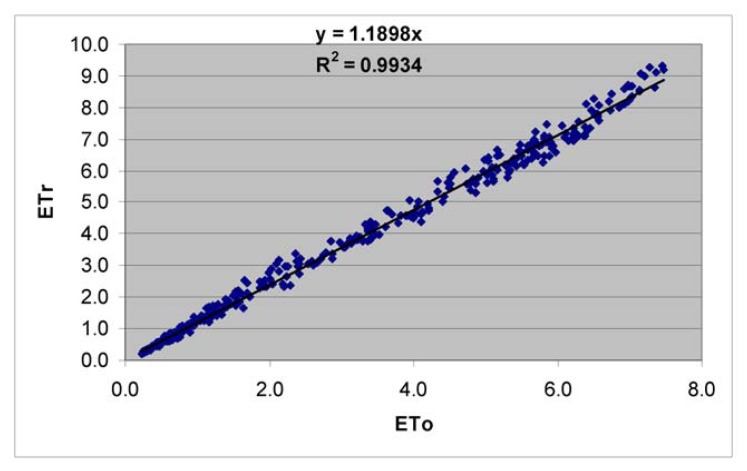
Alfalfa (ETr) versus grass (ETo) reference ET relationship.

The FAO Penman-Monteith formula (Equation 7) was applied for calculation of grass (ETo) and alfalfa (ETr) reference evapotranspiration, by using of daily meteorological data (minimum and maximum air temperature, relative humidity, wind speed, and sun shine duration hours) from the closest (Syrdarya) meteorological station. The correlation between ETr and ETo values were very high (Figure 11).

Because we have monthly satellite images for ET fraction modeling, the average monthly reference ETo values were calculated using daily ETo values, provided in Table 5, multiplied by the number of days in each month during cotton growing period.

#### 6.2.3 Water use (ET_actual_) calculation

**Figure 12.**
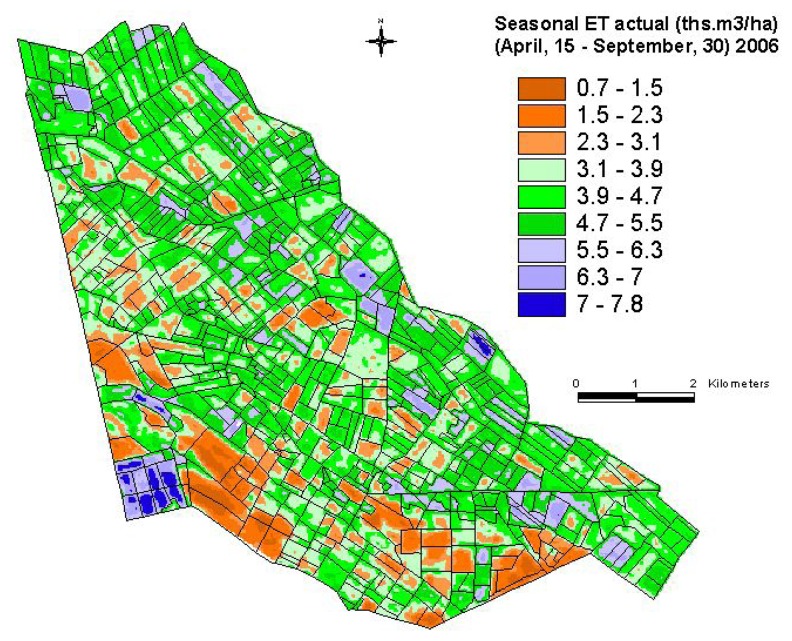
Actual seasonal evapotranspiration (ET_actual_) in the Galaba study area for the cotton growing period of year 2006.

First, the daily water use (m^3^/ha or mm/pixel) was determined by multiplying the reference ET (m^3^/ha or mm/pixel/day) with ET fraction. (dimensionless). Since we have only one image per month, we assumed that the water used for a given day, for which the image was available, remains constant throughout the month for a given crop. Second, the water used per month per crop per pixel is determined leading to accumulation of water use for the pixel over a span of 1 month. Finally, water used by a particular crop for the entire growing season is determined for every pixel. The results are finally presented in thousands m^3^/ha of water used for the entire study area (Figure 12).

### 6.3 Water productivity mapping (WPM)

**Figure 13.**
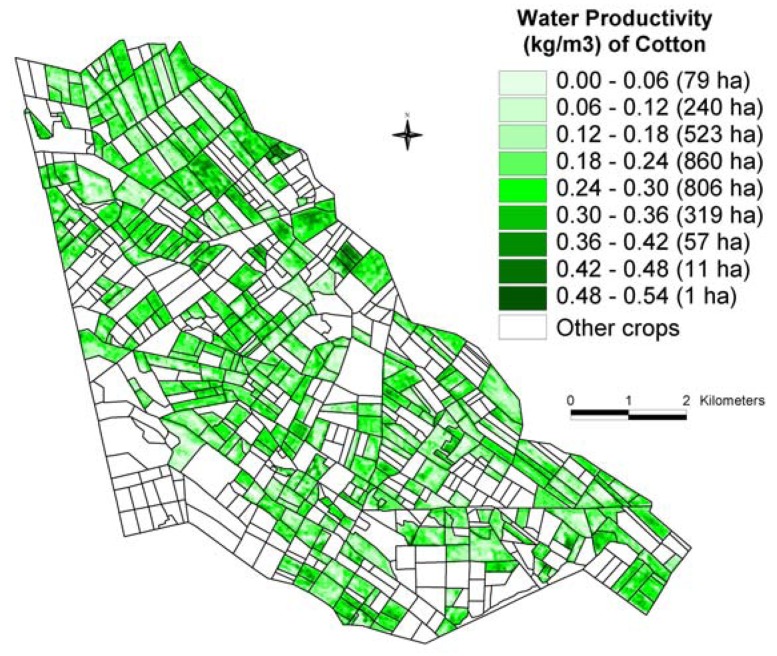
Water prodguctivity map (WPM) of the cotton crop in the Galaba study area.

The crop productivity (ton/ha) raster layer (Figure 7) is divided by seasonal water use (thousands m^3^/ha) raster layer (Figure 12) to obtain water productivity (kg/m^3^) map (WPM), as illustrated for the cotton crop (Figure 13) in Galaba study area. The WPM (Figure 13) shows within and between field variability in crop water productivity. The results in Figure 13 showed that nearly 87% (2,508 hectares) of the total area (2,896 hectares) is in low WP of 0.30 kg/m^3^ or less. This clearly implies the opportunity to grow more food in existing lands through better land and water management practices.

### 6.4 Discussions and validations

The extensive literature review of WP values in the world for main irrigated crops by Zwart et.al. [4] showed a wide range (min – max) of WP (kg/m^3^) for crops: wheat (0.11 – 2.67), rice (0.46 – 2.2), cotton (0.10 – 1.70), and maize (0.22 – 3.99). The highest values of cotton WP (0.54 – 1.70) are reported in Uzbekistan [65] for drip irrigation; but in flooded irrigation by gravity flow as in this study the WP values were between 0.2-1.0 [14, 63, 65]. In this study maximum cotton WP in Galaba farm was 0.54 kg/m^3^; lower values mainly because of the high percentage of soil salinity (43% of farms as determined through field visit in this research) and water logging (31% of farms). The other factors that influenced WP variations were land leveling (14%), water deficit (7%), and others (5%).

**Figure 14.**
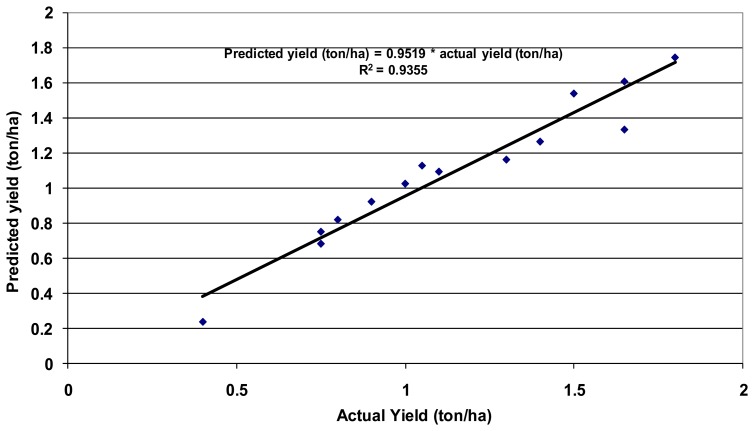
The model was validated using yield data from an independent study [63].

**Figure 15.**
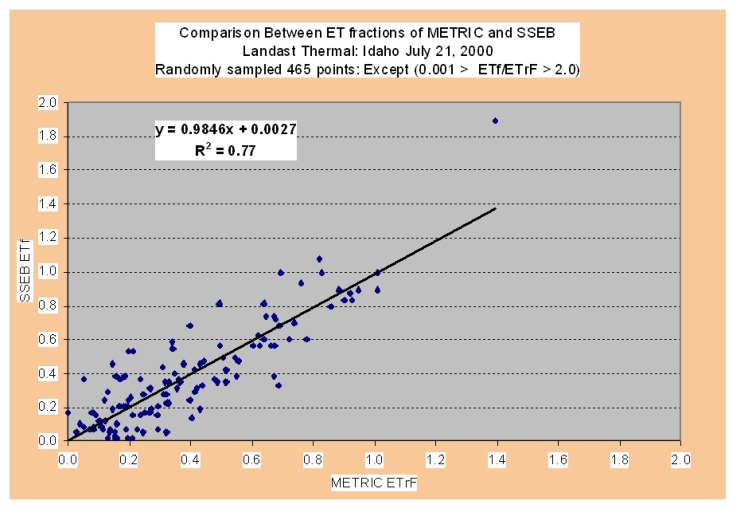
Comparison between ET fraction from METRIC and SSEB models (personal correspondence with Dr. Gabriel Senay developer of SSEB and Dr. Rick Allen developer of METRIC).

We used the yield data from the study [63] to validate the yield model (Figure 14). This re-affirms the validity of the crop productivity maps (CPMs). The water use maps (WUMs) depend on the validity of the ET fraction maps and reference ET maps. The ET fraction computed in this study using SSEB method is known to have very good correlation with ET fraction computed by METRIC (Figure 15). We established this and report these results based on the data provided by the developer of SSEB (Dr. Gabriel Senay) and developer of METRIC (Dr. Rick Allen). The different methods of reference ET calculations do produce slightly different values even when using the same meteorological data (Figure 11). Allen *et al.* [63] reported the ratio of alfalfa (ET_r_) to grass (ETo) reference ET in the range (1.2 – 1.5), our results have shown the similar ratio (1.1898). To get the same actual ET values, applying different methods for reference ET calculation do require using of different crop coefficients.

The ET fraction values (crop coefficients) from SSEB modeling are in the range (0-1), but according to Allen *et al.* [52], maximum Kc for main crops (cotton, rice, wheat and maize) are around (1.15-1.2), it means that seasonal ET can be underestimated and water productivity values overestimated. One solution to overcome it is to use the ET fraction from SSEB with alfalfa (ETr) reference ET, but the final decision does require validation by actual ET by field measurements. The field equipment for actual ET estimation, such as Eddy Systems and Bowen Ratio Towers [53, 64] are very expensive and limited budget did not allow us to validate the result of actual ET modeling by SSEB using data from these towers that did not exist in the region. However, given the confidence with which we have computed crop yield (Figure 14), ET fraction (Figure 15), and reference ET (Figure 11), it can be inferred that the results of this research are reasonable. The emphasis of this study was in development of methods and protocols for WPM using high spatial resolution data. This was achieved through this research.

The need for conducting WPM studies using high spatial resolution remote sensing data from Landsat type sensors is critical so that crop level water use and WP can be studied. This is a significant advantage over coarser resolution imagery such as from MODIS. Nevertheless, the absence of frequent availability of high resolution images is a limiting factor. The SSEB model is very useful in a developing country set up where rigorous data required by METRIC or SEBAL may discourage WPM studies using remote sensing.

## 7. Conclusions

The paper demonstrated the methods and protocols of water productivity mapping (WPM) using high spatial resolution remote sensing data from Landsat-7 ETM+ multi-spectral imagery with thermal bands. The WPMs were produced by first developing crop productivity maps (CPMs) through yield modeling and then dividing them with water use maps (WUMs) through simplified surface energy balance model (SSEB). The outcome was WPMs showing within and between field variations in water productivity (WP), pin-pointing areas of low and high WP, of irrigated crops in the Syrdarya river basin, Central Asia. The main limitations of the study were the absence of: (a) more frequent imagery, and (b) field equipment for actual ET validations.

The cotton crops, which constitute an overwhelming proportion of the study area, showed high variability in WP (0-0.54 kg/m^3^) with 87 % of the cropped area having low WP (< 0.30 kg/m^3^), 11 % area with medium WP (0.30-0.36 kg/m^3^), and only a very small proportion of 2 % having high WP (> 0.36 kg/m^3^). This clearly implies that there is an overwhelming proportion of cropland areas where better management practices of land and water can help to increase WP, thus leading to food security without having to increase allocations of land and water resources.

## Figures and Tables

**Table 1.** Characteristics of Landsat ETM+ data used in this study.

**Acquisition Date**	**Julian Day**	**Sun Elevation**	**Sun Azimuth**	**Earth-Sun distance**

		**(deg.)**	**(deg.)**	**(Astronomic unit)**

2006_0424	114	56.388	138.573	1.005779
2006_0510	131	60.608	134.262	1.010059
2006_0611	162	64.404	125.537	1.015454
2006_0729	210	60.030	128.871	1.015165
2006_0814	226	56.740	134.465	1.012679
2006_1001	274	42.732	152.052	1.000576

**Table 2.** Spectral radiances (LMin/LMax) and mean solar exoatmospheric irradiances (ESUN) for Landsat-7 ETM+ bands.

**Gain**		**Band1**	**Band2**	**Band3**	**Band4**	**Band5**	**Band6**	**Band7**

Low	LMin	-6.2	-6.4	-5.0	-5.1	-1.0	0.0	-0.35
Low	LMax	293.7	300.9	234.4	241.1	47.57	17.04	16.54
High	LMin	-6.2	-6.4	-5.0	-5.1	-1.0	3.2	-0.35
High	LMax	191.6	196.5	152.9	157.4	31.06	12.65	10.80

ESUN_λ_		1969	1840	1551	1044	225.7		82.07

**Table 3.** Field-plot data characteristics of various variables for different crops.

**Crop**	**Number of samples**	**Day of Year**	**Mean values from the samples**			
			**Wet biomass**	**Dry biomass**	**Leaf Area Index**	**Crop Yield**
			**(kg/m**^**2**^**)**	**(kg/m**^**2**^**)**	**(m**^**2**^**/m**^**2**^**)**	**(ton/ha)**
Wheat	28	127	1.69	0.4	2.16	1.850
		145	1.33	0.65	2.17	
		158	0.65	0.43	1.32	
Cotton	162	127	0.02	0.00	0.07	1.230
		145	0.04	0.01	0.28	
		158	0.12	0.02	0.49	
		173	0.25	0.05	0.60	
		188	0.88	0.23	1.78	
		200	1.25	0.26	2.38	
		214	1.22	0.35	2.04	
		229	1.52	0.62	2.53	
		247	3.05	1.41	1.71	
		256	3.09	1.36	1.87	
		271	1.90	1.06	1.40	
Rice	43	173	0.29	0.04	0.65	4.315
		188	0.75	0.24	1.59	
		200	1.02	0.24	1.24	
		214	1.54	0.80	2.12	
		229	1.91	0.72	5.56	
		247	3.49	1.58	5.60	
		256	4.41	2.18	3.24	
		271	1.17	0.65	1.24	
Maize	40	173	0.02	0.00	0.19	3.305
		188	0.04	0.01	0.25	
		200	0.07	0.01	0.47	
		214	0.37	0.07	0.87	
		229	1.05	0.48	0.99	
		247	3.63	1.90	1.48	
		256	3.25	1.74	1.52	
		271	3.09	1.98	1.05	

**Table 4.** Values of coefficients for reference ET calculation.

**Calculation time step**	**Short Reference (ETo)**		**Tall Reference (ETr)**	

	**(clipped grass)**		**(alfalfa)**	

	**Cn**	**Cd**	**Cn**	**Cd**

Daily	900	0.34	1600	0.38
Hourly during daytime	37	0.24	66	0.25
Hourly during nighttime	37	0.96	66	1.7

**Table 5.** Monthly ETo (mm/day) and number of days in each month for cotton growing.

	**Jan**	**Feb**	**Mar**	**Apr**	**May**	**Jun**	**Jul**	**Aug**	**Sep**	**Oct**	**Nov**	**Dec**

Cotton				15	31	30	31	31	30			
ETo	0.6	0.944	1.753	3.398	5.081	6.716	7.066	6.269	4.466	2.587	1.103	0.656
